# Hydroxyurea interferes with point-of-care creatinine testing in children with sickle cell anemia

**DOI:** 10.1186/s13104-026-07916-1

**Published:** 2026-06-19

**Authors:** Ruth Namazzi, Robert O. Opoka, Charles Ssuuna, Michael J. Goings, Kathryn McElhinney, Dibyadyuti Datta, Russell E. Ware, Chandy C. John, Andrea L. Conroy

**Affiliations:** 1 Department of Pediatrics and Child Health, Makerere University College of Health Sciences, Kampala, Uganda; 2 Department of Pediatrics and Child Health, The Aga Khan University, Nairobi, Kenya; 3 CHILD laboratory, Global Health Uganda, Kampala, Uganda; 4 Ryan White Center for Pediatric Infectious Disease and Global Health, Indiana University School of Medicine, R4 402C 1044 West Walnut St, Indianapolis, IN 46202 USA; 5 Department of Pediatrics, Division of Hematology, Cincinnati Children’s Hospital Medical Center, Cincinnati, OH USA; 6 University of Cincinnati College of Medicine, Cincinnati, OH USA

**Keywords:** Creatinine, Hydroxyurea, Interference, Sickle cell anemia, Point-of-care testing, Diagnostic accuracy

## Abstract

**Objective:**

To evaluate the performance of the point-of-care i-STAT device for creatinine measurement in Ugandan children with sickle cell anemia (SCA), and to determine the clinical impact of hydroxyurea-associated assay interference.

**Results description:**

This secondary analysis was nested within a randomized clinical trial involving 248 Ugandan children with SCA. The mean age at enrollment was 32 months, and 115/248 (46.2%) initiated hydroxyurea during 12 months of follow-up as part of routine clinical care. At enrollment, creatinine values measured by i-STAT and reference laboratory testing were clinically comparable. At follow-up, children receiving hydroxyurea had significantly higher creatinine values measured by i-STAT than by reference laboratory testing (mean difference + 0.19 mg/dL; *p* = 0.006), with several i-STAT results falling within the clinically abnormal range. No such discrepancy was observed among children not receiving hydroxyurea. In a subset of samples, serum hydroxyurea concentrations measured by high-performance liquid chromatography accounted for 73% of the variability in i-STAT creatinine values. These findings demonstrate clinically meaningful interference of hydroxyurea with enzymatic point-of-care creatinine testing, which may result in misclassification of kidney function and inappropriate clinical decision-making in children with SCA receiving hydroxyurea. ClinicalTrials.gov identifier: NCT03528434 (date registered: May 7^th^, 2018).

**Supplementary Information:**

The online version contains supplementary material available at https://doi.org/10.1186/s13104-026-07916-1.

## Introduction

The ability to evaluate kidney function is essential for identifying acute and chronic kidney disease and for monitoring medication safety. Serum creatinine remains the most widely used and accessible marker for estimating glomerular filtration rate (eGFR) globally [1] to screen for kidney disease in high-risk populations [2]. However, in many low-income settings, creatinine testing is centralized at referral hospitals or private laboratories, limiting timely and equitable access to kidney function assessment, particularly for children with chronic conditions [3].

Point-of-care (POC) testing platforms offer an attractive strategy for decentralizing laboratory testing and expanding access to essential diagnostics. The i-STAT system (Abbott Point of Care Inc.) is widely used for POC creatinine measurement and has been adopted in both high- and low-resource settings. Children with sickle cell anemia (SCA) represent a population in whom reliable kidney function monitoring is especially important, given their increased risk of both acute kidney injury and chronic kidney disease, as well as the need for routine laboratory monitoring during disease-modifying therapy.

In the present study, we evaluated creatinine measurements obtained using the i-STAT POC device in a cohort of young children with SCA initiated on hydroxyurea as a disease-modifying therapy [4]. Although hydroxyurea is listed as an interfering substance for i-STAT enzymatic creatinine assays, this limitation may not be widely recognized in routine clinical practice. While it is described in manufacturer documentation and analytical evaluations, its clinical relevance in pediatric populations and real-world settings has not been well characterized. Given the increasing use of i-STAT devices in low-resource settings where hydroxyurea is becoming standard-of-care therapy for children with sickle cell anemia, understanding the magnitude and clinical consequences of this interference is increasingly important. Front-line clinicians relying on point-of-care creatinine testing may not be aware of the potential for hydroxyurea-associated assay interference. This analysis was prompted by observed discrepancies between point-of-care and reference creatinine measurements during participant follow-up and was undertaken to evaluate the magnitude and clinical implications of hydroxyurea-associated assay interference in this setting. We observed clinically significant discrepancies between POC and reference laboratory creatinine values among children receiving hydroxyurea, raising concern that unrecognized assay interference could erroneously prompt changes in hydroxyurea treatment or other aspects of clinical management.

## Methods

Children were enrolled in a randomized double-blind placebo-controlled, parallel group superiority trial with a 1:1 allocation ratio evaluating daily oral zinc supplementation (10 mg elemental zinc) for infection prevention in children with SCA in Jinja, Uganda [4]. The placebo-controlled component of the parent trial applied only to zinc supplementation; hydroxyurea was offered as standard-of-care therapy and was not randomized. The trial protocol, statistical analysis plan, and primary results are publicly available [4, 5]. Inclusion criteria included documented SCA (HbSS or HbS/β° thalassemia confirmed by hemoglobin electrophoresis using standard clinical laboratory methods, including quantitative hemoglobin fractionation to distinguish HbSS from HbS/β°-thalassemia), age 1.00 − 4.99 years at enrollment, weight ≥ 5.0 kg at enrollment, and a willingness to comply with all study-related treatments, evaluations, and follow-up. Exclusion criteria included known chronic medical condition (HIV infection, malignancy, or active clinical tuberculosis) or severe malnutrition defined using World Health Organization growth standards (weight-for-height < − 3) [4]. The trial was registered on ClinicalTrials.gov, NCT03528434 (date registered: May 7th, 2018). As part of routine clinical care, children were initiated on hydroxyurea as a disease-modifying therapy for SCA at approximately 20 mg/kg/day in accordance with Uganda Ministry of Health guidelines. Dose escalation was not protocol-driven within the study, and capsules were opened and administered as dispersed formulations for young children [6].

At enrollment and at the 12-month follow-up visit, all children underwent venous blood collection for clinical tests, including kidney function assessments. Malaria testing was not routinely performed at scheduled study visits unless clinically indicated, as visits were conducted during routine outpatient follow-up visits rather than acute febrile illness. Serum was collected using additive-free tubes and stored at − 80 °C until batch testing at the Mulago Hospital Chemistry Laboratory using an isotope dilution mass spectrometry (IDMS)-traceable creatinine assay using the modified Jaffe method (Cobas c311). The primary outcome of this secondary analysis was the difference between point-of-care i–STAT creatinine values and reference laboratory creatinine values at enrollment and 12-month follow-up. Secondary outcomes included agreement between measurement methods assessed by Bland-Altman analysis and the association between serum hydroxyurea concentrations and i-STAT creatinine values. No formal sample size calculation was performed for this secondary analysis. All available participants with paired point-of-care and reference laboratory creatinine measurements were included.

Point-of-care creatinine testing was conducted on whole blood using the point-of-care CHEM8 + or Crea cartridge on the i-STAT handheld blood analyzer (Abbott Point of Care Inc., Princeton, NJ). The handheld i-STAT system measures creatinine using an enzymatic assay. Creatinine values are traceable to the U.S. National Institutes of Standards and Technology (NIST) Standard Reference material SRM909. The reportable range for serum creatinine is 0.20–20.0 mg/dL, and samples below the detection limit were assigned a value of 0.19 mg/dL.

Because hydroxyurea is listed as an interfering substance for creatinine measurement on the i-STAT platform, participants were counselled to delay taking hydroxyurea until after the scheduled blood draw. In a subset of samples with large discrepancies in point-of-care and reference laboratory creatinine results, high-performance liquid chromatography was used to quantify hydroxyurea levels in serum. These samples were selected based on observed discrepancies i.e. all samples with a difference of > 0.1 (i-Stat - reference). We then selected 8 controls from those with a difference < − 0.1. The only exclusions from this analysis were two participants already receiving hydroxyurea at enrollment.

Data were analyzed using STATA v14.0 (StataCorp) and GraphPad Prism v7.03. Analyses were conducted using available data only. No imputation was performed for missing creatinine or hydroxyurea concentration data. Differences in creatinine measures by test methodology were evaluated. Concentrations of creatinine were compared using Pearson’s correlation. The Bland-Altman method was used to measure agreement between the creatinine values obtained by each test approach based on the variance of differences in concentration across the mean of concentration values [7]. Bias was represented by the mean difference between the reference and i-STAT creatinine, and precision was defined as one standard deviation of the bias.

## Results and discussion

**Fig. 1:**
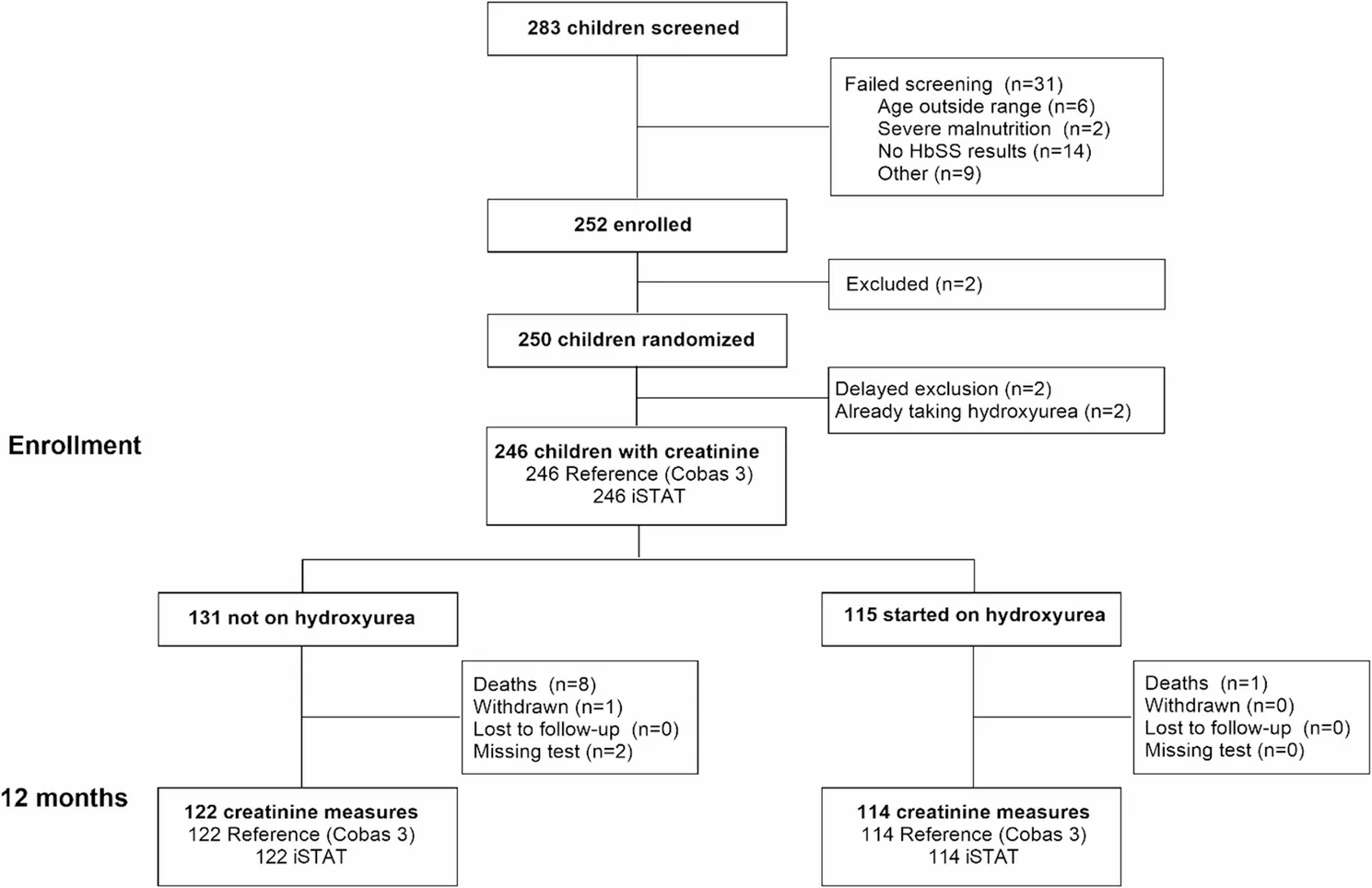
Flow chart of study population

The trial completed follow-up as planned, with no early termination or stopping for benefit, harm, or futility. A total of 248 children were enrolled in the study from March 2019 to November 2020 and were actively followed for 12 months. Baseline demographic and clinical characteristics for participants have been previously reported in the parent ZIPS trial publication [4]. The mean age of children at enrollment was 32.5 months (standard deviation, 13.6). A flow chart of the study population is shown in Fig. 1. Only 2 of the 248 children were on hydroxyurea treatment at enrollment. Parents of the enrolled children were strongly encouraged to have their children receive hydroxyurea treatment at the standard Uganda Ministry of Health dosing of 20 mg/kg/day. Hydroxyurea treatment was paid for by the study. Over the course of the study, parents of 115 of the 248 children (46.2%) agreed to have their child started on hydroxyurea treatment. Baseline creatinine values measured by i-STAT were comparable between children later initiated on hydroxyurea and those who were not (0.21 mg/dL [SD 0.05] vs. 0.22 mg/dL [SD 0.06], *p* = 0.14).

At enrollment, the mean creatinine level by i-STAT was 0.22 mg/dL (SD, 0.05). At 12-month follow-up, several children had a clinically significant increase in serum creatinine measured with i-STAT, with values exceeding 1.0 mg/dL and reaching as high as 5.0 mg/dL. Samples were retested at an external laboratory, and levels were comparable to those at enrollment. Subsequent investigations revealed that the samples flagged for increased creatinine levels were from children receiving hydroxyurea.

All samples from study participants were then retested using a reference laboratory using an IDMS-traceable assay. At enrollment, the mean creatinine was 0.30 mg/dL (SD, 0.06) at the reference laboratory using a modified Jaffe assay compared to 0.22 mg/dL (SD, 0.05) by the enzymatic i-STAT assay, corresponding to a clinically comparable but modestly higher reference laboratory value compared to i-STAT (mean difference + 0.08 mg/dL, SD 0.06; paired t-test, *p* < 0.0001), correlation coefficient of 0.42 (*p* < 0.0001).

**Fig. 2:**
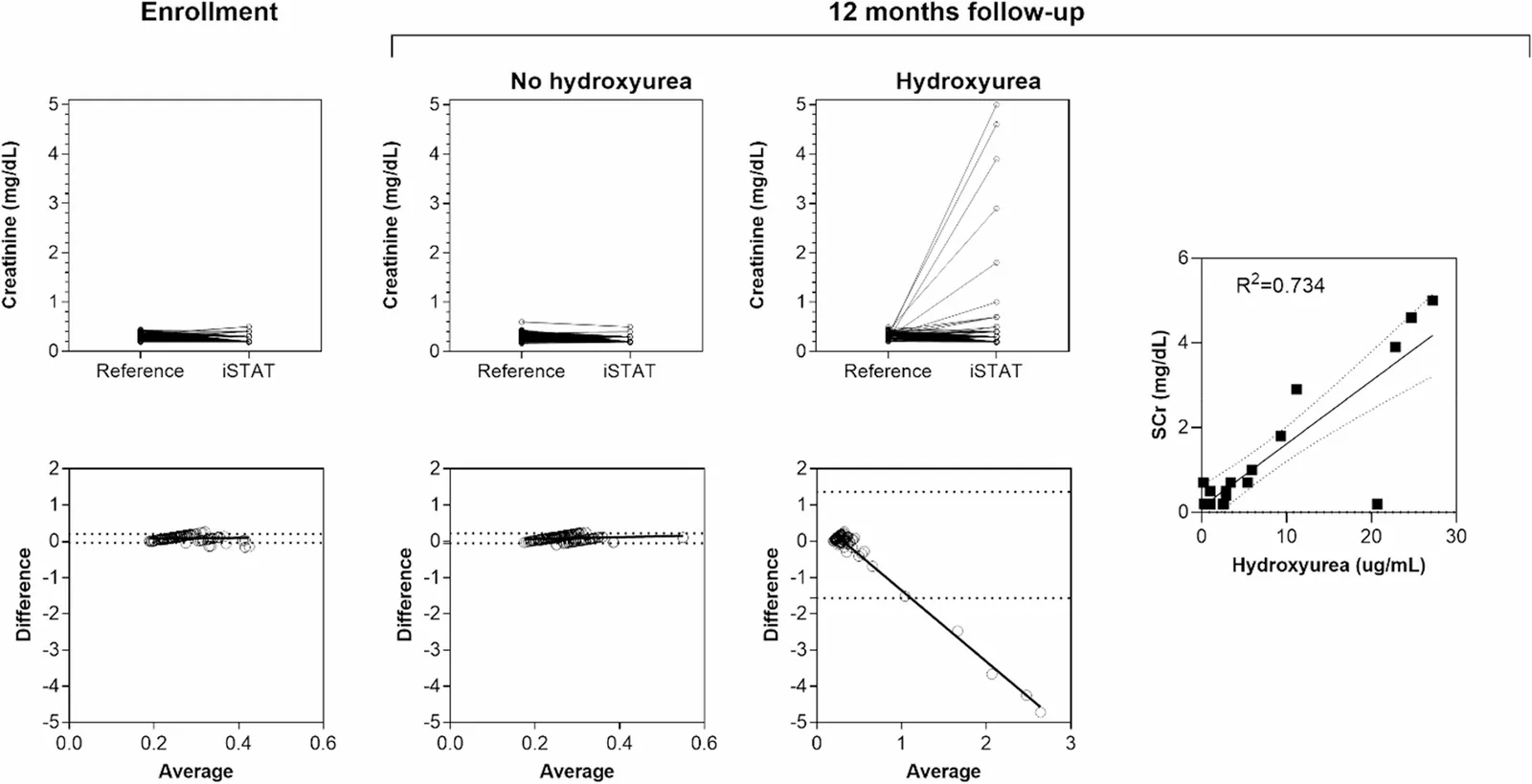
Creatinine levels in children with sickle cell anemia at enrollment and at 12-month follow-up based on hydroxyurea use. Top row. Line graph comparing creatinine measures in children at enrollment and 12 months follow-up based on hydroxyurea use. Bottom row. Bland Altman analysis comparing the difference in creatinine measures using the reference test and iSTAT test in samples collected at enrollment and 12 months follow-up. Right. Hydroxyurea concentrations were measured in plasma samples from 20 participants at 12-month follow-up to quantify the impact of hydroxyurea concentrations in the blood on creatinine measures

When comparing values from enrollment to 12-month follow-up, the reference laboratory values were similar, with creatinine levels of 0.30 (SD, 0.06) at enrollment and 0.31 (SD, 0.06) at 12-month follow-up (*p* = 0.03 by paired t-test). When comparing the difference in levels between enrollment and 12-month follow-up by i-STAT, the difference was pronounced with a mean of 0.22 (0.06) at enrollment and 0.32 (0.52) at 12-month follow-up (*p* = 0.004 by paired t-test). At the 12-month follow-up visit, the mean difference between the reference laboratory result and i-STAT was comparable to the difference observed at enrollment in children not receiving hydroxyurea (reference laboratory 0.30 mg/dL (SD, 0.06) vs. i-STAT 0.23 (SD, 0.06), mean difference + 0.08 mg/dL, SD 0.07; paired t-test, *p* < 0.0001), correlation coefficient of 0.34 (*p* = 0.0001). In contrast, among children receiving hydroxyurea, iSTAT creatinine values were higher than the reference laboratory values (i-STAT 0.41 mg/dL (SD, 0.75) vs. reference laboratory, 0.31 mg/dL (SD, 0.06), *p* < 0.0001) with a mean difference of − 0.10 mg/dL (SD, 0.75) and no significant correlation between test modalities (correlation coefficient 0.03, *p* = 0.734) (Fig. 2).

Subsequent analysis compared serum creatinine values with hydroxyurea concentrations in stored samples, measured by HPLC. Use of i-STAT creatinine measurements resulted in substantially more children meeting criteria for abnormal estimated glomerular filtration rate among those receiving hydroxyurea compared with reference laboratory testing, supporting the potential for clinically significant kidney function misclassification. While there was no correlation between hydroxyurea concentrations and creatinine levels in the reference laboratory (Pearson *r* = 0.048, *p* = 0.46), there was a strong correlation between serum hydroxyurea concentrations and creatinine values measured by i-STAT (Pearson *r* = 0.855, *p* < 0.001) (Fig. 2). A single outlier was retained in the analysis and did not materially alter the observed relationship. It may reflect variability in pharmacokinetics or timing of hydroxyurea dosing relative to blood sampling.

Our study reveals important considerations regarding the use of point-of-care (POC) creatinine testing with the i-STAT device in children with SCA undergoing hydroxyurea therapy. POC devices like the i-STAT offer decentralization and rapid results that can enhance monitoring in high-risk populations, including children with SCA who require routine kidney function assessment during hydroxyurea treatment.

However, our findings demonstrate a significant and clinically meaningful interference of hydroxyurea with creatinine measurements performed by the i-STAT device, consistent with previous reports documenting falsely elevated creatinine and glucose readings in the presence of hydroxyurea using similar enzymatic and electrochemical assay platforms [8]. The observed relationship between hydroxyurea concentrations and discrepant i-STAT creatinine values indicates that hydroxyurea is a major contributor to measurement bias in this setting. Zinc supplementation, administered at 10 mg daily in the parent trial, was not associated with i-STAT creatinine levels in exploratory analyses and is not known to interfere with hydroxyurea pharmacokinetics or enzymatic creatinine assays. This interference likely arises from the electrochemical detection mechanisms used in the i-STAT CHEM8 + or Crea cartridges, as hydroxyurea and related metabolites may generate signals indistinguishable from creatinine [9].

Clinical incidents have been reported in which erroneous POC creatinine elevations prompted inappropriate management decisions, including unnecessary hospital admissions and treatment alterations [10]. The implications for clinical practice are significant. In this context, unrecognized hydroxyurea-related assay interference could lead to misclassification of kidney function and inappropriate changes in hydroxyurea therapy or other aspects of clinical management. As children with SCA are at risk of both acute kidney injury and chronic kidney disease, accurate interpretation of creatinine measures is of critical importance [11]. Although malaria is endemic in the study setting, samples were collected during routine outpatient visits rather than acute illness, making active malaria infection an unlikely explanation for the observed discrepancies. These findings differ from prior studies in children with severe malaria, where i-STAT underestimated creatinine, likely reflecting differences in clinical context and the presence of hydroxyurea exposure in the current study. In the prior severe malaria cohort, i-STAT testing underestimated creatinine relative to reference testing, with minimal bias, whereas in the current cohort, hydroxyurea exposure was associated with elevated i-STAT creatinine values and a reversal of the bias.

Counseling patients to withhold hydroxyurea before sampling, as done in this study, may reduce but not eliminate interference, given the variability in drug pharmacokinetics and adherence. Alternative strategies include confirmatory testing using standard laboratory enzymatic assays traceable to IDMS standards, or employing POC devices validated to have minimal hydroxyurea interference, although such options are often limited in resource-constrained settings.

Our findings highlight the critical need for clinicians and laboratory personnel to be aware of this assay limitation, incorporate medication histories when interpreting POC creatinine results, and advocate for assay improvements or alternative monitoring strategies suitable for children with SCA on hydroxyurea. This is relevant not only for routine outpatient monitoring but also for inpatient care and hospitalizations associated with an increased risk of acute kidney injury [11].

While POC creatinine testing with the i-STAT device offers clear advantages for decentralizing kidney function monitoring in resource-limited environments, hydroxyurea interference limits its reliability in children with SCA receiving this therapy. Future efforts should prioritize clinician education, assay refinement, and the integration of confirmatory laboratory testing when feasible to ensure accurate assessment of kidney function and optimal management in this key population.

## Study limitations

The trial was not initially designed to evaluate assay interference, as participants were counseled not to take hydroxyurea before their study visit. Detailed characterization of disease severity and hydroxyurea response phenotypes was not performed, limiting assessment of clinical heterogeneity. Hydroxyurea concentrations were only measured in a subset of participants with evidence of assay interference. Adherence to instructions to delay hydroxyurea dosing prior to blood collection was not formally measured and may have contributed to variability in observed interference. Clinical factors such as hydration status, intercurrent illness, transfusions, and concomitant medications were not systematically captured and may have contributed to variability in creatinine measurements. However, since we are comparing differences in creatinine levels at the same time point within a participant between i-STAT and a clinical chemistry machine, there is no reason to think that hydration status or other factors would differentially affect the assay. Previous studies demonstrated minimal baseline bias between i-STAT and reference creatinine assays in children not receiving hydroxyurea, supporting the interpretation that the observed discrepancies are primarily attributable to hydroxyurea-associated assay interference.

## Supplementary Information


Supplementary Material 1.


## Data Availability

The datasets used and/or analysed during the current study are available from the corresponding author on reasonable request.
